# Retrospective analysis of computed tomography-guided percutaneous nephrostomies in cancer patients

**DOI:** 10.1590/0100-3984.2018.0023

**Published:** 2019

**Authors:** Marcio dos Santos Meira, Paula Nicole Vieira Pinto Barbosa, Almir Galvão Vieira Bitencourt, Maria Fernanda Arruda Almeida, Chiang Jeng Tyng, Maria Alice Freitas Costa, Ana Carolina de Ataíde Góes, Rubens Chojniak

**Affiliations:** 1 Department of Imaging, A.C.Camargo Cancer Center, São Paulo, SP, Brazil.

**Keywords:** Nephrostomy, percutaneous/methods, Nephrostomy, percutaneous/adverse effects, Computed tomography, Nefrostomia percutânea/métodos, Nefrostomia percutânea/efeitos adversos, Tomografia computadorizada

## Abstract

**Objective:**

To establish an overview of computed tomography (CT)-guided percutaneous nephrostomy performed at a referral center for cancer, addressing the characteristics of patients submitted to this intervention, as well as the indications for it, the technical specificities of it, and its main complications.

**Materials and Methods:**

This was a retrospective study involving a review of the electronic medical records and images of patients submitted to CT-guided percutaneous nephrostomy at a referral center for cancer between 2014 and 2016.

**Results:**

A total of 201 procedures were evaluated. In most cases, the obstruction was caused by a malignant neoplasm. Complications occurred in 9.5% of the cases, and an additional intervention was required (typically for catheter repositioning) in 36.6%. Post-procedure complications were not found to be significantly associated with the type of previous cancer treatment, the technique used, the caliber of the drain used in the procedure, or the degree of dilatation of the collection system prior to the procedure.

**Conclusion:**

In cancer patients, CT-guided percutaneous nephrostomy is an effective treatment, with success rates and complication rates similar to those reported in the general population.

## Abstract (pt)

**Objetivo:**

Estabelecer o perfil da nefrostomia percutânea guiada por tomografia computadorizada (TC) em um centro de referência oncológico, conhecendo as características do paciente submetido a esta intervenção, indicações e especificidades técnicas do procedimento, além das complicações mais frequentes.

**Materiais e Métodos:**

Foi realizada revisão dos prontuários eletrônicos e das imagens de pacientes submetidos a nefrostomia percutânea guiada por TC entre os anos de 2014 e 2016 em um centro de referência oncológico.

**Resultados:**

Foram avaliados 201 procedimentos. As doenças neoplásicas malignas foram as principais causas da obstrução. Houve necessidade de reabordagem em 36,6% dos casos, em sua maioria para reposicionamento do cateter, e ocorreram complicações em 9,5% dos casos. Não houve associação estatisticamente significante entre as complicações pós-nefrostomia percutânea e o tipo de tratamento oncológico prévio, a técnica empregada, o calibre do dreno utilizado no procedimento, ou o grau de dilatação do sistema coletor prévio ao procedimento.

**Conclusão:**

A nefrostomia percutânea guiada por TC é eficaz em pacientes oncológicos, com taxas de sucesso e complicações semelhantes às observadas na população geral na literatura.

## INTRODUCTION

Percutaneous nephrostomy is a procedure, typically performed by interventional radiologists, that aims to provide temporary or permanent alternative drainage of the upper urinary tract, as a solution to mechanical obstruction or other defects of the drainage system not resulting in direct occlusion^(^^1^^-^^3^^)^. Percutaneous nephrostomy is a well-established practice. The use of imaging modalities to guide the procedure and improvements in the equipment employed have made it possible to perform novel techniques, resulting in a reduction in the associated morbidity and broadening the indications for the procedure.

The classic objectives of percutaneous nephrostomy include the following: to dissolve calculi; to infuse chemotherapeutic, antibiotic, or antifungal agents; to divert the renal collecting system in order to optimize the treatment of fistulas resulting from trauma, iatrogenic injury, malignant neoplasms, or inflammatory diseases; to treat complications related to renal transplantation; to extract a foreign body^(^^4^^)^; and to treat other urologic diseases^(^^5^^)^. In the context of cancer treatment, percutaneous nephrostomy plays an important role because it is an effective method of creating an alternative path of urinary drainage to bypass obstructions, most of which are caused by neoplasms in the cervix, prostate, or bladder^(^^6^^)^. Because of its minimally invasive nature, percutaneous nephrostomy guided by axial imaging methods is associated with lower morbidity and less patient discomfort than is the corresponding surgical procedure. Another advantage is that it can be performed with only local anesthesia, with or without conscious sedation, thus avoiding the need for general anesthesia.

The clinical success of percutaneous nephrostomy depends on a number of variables: the biotype and general health status of the patient; the anatomical position and size of the kidney; and the degree of dilatation of the renal pelvis and calyx. In the scientific literature of Brazil, there are no recent studies evaluating the success rate, complication rate, or average durability of percutaneous nephrostomy procedure. Therefore, the present study aimed to provide a profile of percutaneous nephrostomy guided by computed tomography (CT) at a referral center for cancer in Brazil, describing the characteristics of the patients submitted to this intervention, the indications for the procedure, its technical specificities, and the associated complications.

## MATERIALS AND METHODS

Prior to the initiation of data collection, the study was approved by the research ethics committee of the institution. Because this was a retrospective study, based on the review of image examinations and medical records, the requirement for informed consent was waived. We used the information collected solely and exclusively for the execution of the present study, preserving the anonymity of the research subjects whose data were collected.

This was a retrospective study involving patients submitted to CT-guided percutaneous nephrostomy between June 2014 and November 2016. We reviewed electronic medical records, descriptive post-procedure reports, medical reports, and images related to the procedures.

To collect data from medical charts and reports, we used a standardized questionnaire covering the following items: patient chart number, gender, and age; cause of urinary tract obstruction; treatment of the underlying disease; date of the procedure; technique employed; caliber of the drain used; degree of hydronephrosis; complications; and the need for additional intervention. The degree of hydronephrosis was classified as follows^(^^7^^-^^9^^)^: grade 0 (no dilatation of the renal pelvis); grade 1 (very mild-dilatation of the renal pelvis without dilatation of the renal calyces or atrophy of the renal parenchyma); grade 2 (mild-minimal dilatation of the renal pelvis and calyx, without parenchymal atrophy); grade 3 (moderate-moderate dilatation of the renal pelvis and calyces, with flattening of the papillae, with or without minimal atrophy of the parenchyma); or grade 4 (severe-marked dilatation of the renal pelvis and calyces, as well as significant atrophy of the parenchyma).

The information collected with the questionnaire was exported to a Microsoft Excel spreadsheet. The IBM SPSS Statistics software package, version 20.0 (IBM Corp., Armonk, NY, USA) was used for data processing. We calculated descriptive statistics, adopting the usual measures of central tendency (mean, median, and mode) and dispersion (range, variance, standard deviation, and coefficient of variation), together with absolute and relative frequencies. When necessary, statistical tests were applied in order to detect correlations between the variables: the chi-square test and Fisher's exact test, for correlations between categorical variables; Student's t-test for correlations between continuous variables with normal distribution; and the Mann-Whitney test, for correlations between continuous variables with non-normal distribution. The level of significance was set at 5%.

### Standardized interventional radiology protocol for percutaneous nephrostomy procedures

The pre-procedure evaluation includes the following: patient anamnesis, directed at the investigation of comorbidities, allergies, and medications in use, especially anticoagulants and antiplatelet agents; and a review of the coagulation profile and complete blood count, the inclusion criteria being an international normalized ratio < 1.5, a platelet count > 50,000/mm^3^, and hemoglobinemia (hemoglobin > 7.0 mg/dL).

All pre-procedure data are recorded on a mandatory institutional form. For all procedures, the patient or a legal guardian gives written informed consent.

Prior to the procedure, patients should fast for six hours (for conscious sedation) or eight hours (for general anesthesia).

In all patients, calibrated peripheral venous access is obtained and venous hydration is maintained. Approximately 30 min before the procedure, antibiotic prophylaxis is administered intravenously-with 1-2 g of ceftriaxone or (in the case of penicillin allergy) with 600 mg of clindamycin plus 5 mg/kg of gentamicin. The caliber of the drain routinely used is 8 Fr or 10 Fr, although a 12 Fr drain is preferred in cases of sepsis due to urinary tract infection or clots in the renal pelvis, as well as for catheter exchange (when there is low output or pericatheter extravasation) or to guide urological procedures.

Relevant ultrasound or CT images are reviewed to assess the degree of hydronephrosis and the anatomical position of the kidney in relation to the colon, liver, and spleen, both of which are important factors affecting the choice of the ideal approach.

Percutaneous nephrostomy can be guided by ultrasound^(^^10^^)^, CT, or conventional fluoroscopy^(^^11^^)^. At our center, the main aspects evaluated in order to determine which imaging method will be used in guiding the procedure are the degree of hydronephrosis, patient biotype, patient cooperativeness, and the presence of bleeding disorders. In cooperative patients with dilatation of the renal collecting system (subjectively assessed by an interventional radiologist as moderate or pronounced) and an appropriate biotype (lean patients or those with minimal abdominal adipose tissue), ultrasound guidance is usually chosen. However, in patients with obesity, bleeding disorders or mild dilatation of the collecting system, we generally choose CT guidance. This paper describes CT-guided percutaneous nephrostomy.

**Figure 1:**
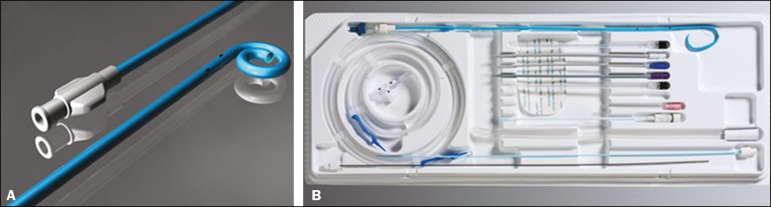
Materials used for percutaneous nephrostomies. **A:** Skater pigtail catheter (10 Fr × 25 cm), for when the trocar technique was chosen. **B:** Nephrostomy kit with a Neo-Hydro hydrophilic drainage catheter, for when the Seldinger technique was indicated.

The following materials were used for percutaneous nephrostomy (Figure 1): a drainage catheter with a locking pigtail (Skater 10 Fr × 25 cm; Argon Medical Devices, Athens, TX, USA) when the trocar technique was chosen; and a nephrostomy kit with a hydrophilic drainage catheter (Neo-Hydro; Bioteque Corp., Taipei, Taiwan) when the Seldinger technique was indicated.

**Figure 2:**
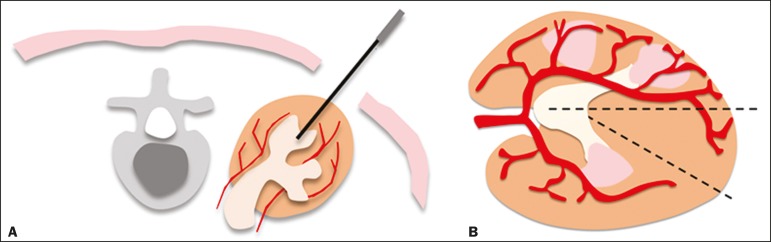
Schematic drawing of the avascular plane, also known as Brödel's line^(^^13^^,^^14^^)^. **A:** Axial slice obtained with the patient in the prone position, demonstrating the ideal entry point for the percutaneous nephrostomy. **B:** Magnification of the angle of entry of the needle into the right kidney, with the patient in the supine position.

**Figure 3:**
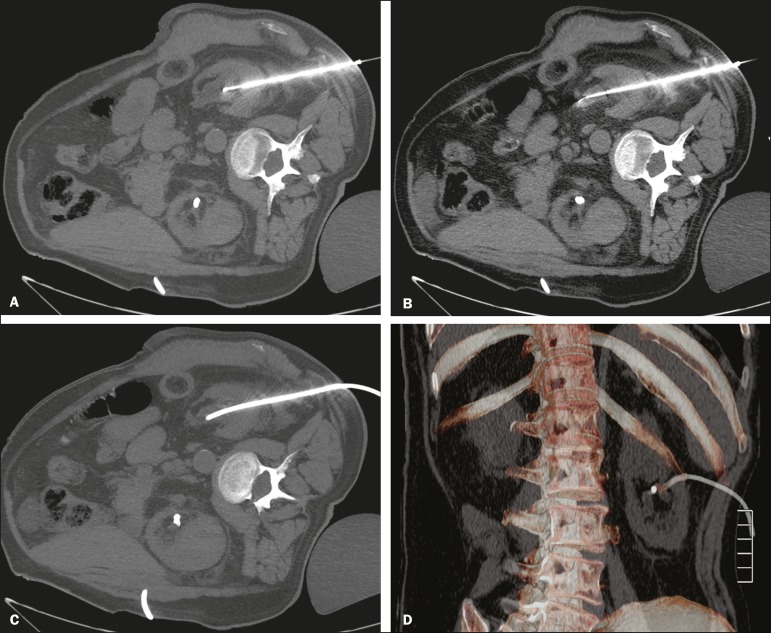
Seldinger technique for percutaneous nephrostomy. **A:** Puncture needle inserted into the renal pelvis. **B:** Guide wire in the lumen of the renal collecting system. **C:** Introduction of the catheter coupled to a rigid rod. **D:** Three-dimensional CT reconstruction of the abdomen showing the nephrostomy catheter in the left kidney, with its end in the renal pelvis.

The procedure is performed with an aseptic technique. Choosing the puncture site is crucial to minimizing the risk of bleeding. The best route for the introduction of the needle into the renal collection system is by oblique posterolateral approach along the avascular plane (Brödel's line), at the level of the posterior renal calyx (Figure 2), corresponding to the zone of lowest vascular density of the renal parenchyma and therefore associated with a lower risk of substantial vascular injury and subsequent bleeding^(^^12^^)^. That access route is commonly possible by puncture at the posterior axillary line, 2-3 cm below the 12th rib (Figure 3).

## RESULTS

**Figure 4:**
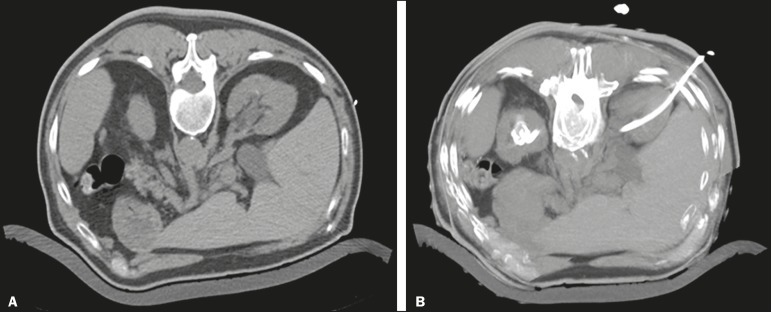
Percutaneous nephrostomy of the right kidney after removal of double J stent. **A:** Pre-procedure axial slice showing slight dilatation of the renal collecting system. **B:** Final follow-up CT, with a maximum intensity projection reconstruction.

A total of 201 procedures were evaluated, of which 116 (57.7%) were performed in men and 85 (42.3%) were performed in women. The mean age of the patients was 63.8 years. The cause of the obstruction was known in 129 (64.2%) cases. Among those 129 cases, the cause of the obstruction was malignant neoplastic disease in 117 (90,7%) and a benign lesion in 12 (9,3%). The degree of hydronephrosis was classified as grade 1 in 15 (7.5%) of the 201 procedures (Figure 4), grade 2 in 50 (24.9%), grade 3 in 47 (23.4%), and grade 4 in 18 (9.0%).

The puncture technique most often used was the Seldinger technique, which was applied in 140 (69.7%) of the procedures. In 16 cases (8.0%), the technique employed was not noted or the procedure was contraindicated. The caliber of the drain used was 10 Fr in 140 patients (69.7%), 12 Fr in 40 (19.9%), 6 Fr in 1 (0.5%), and 14 Fr in 1 (0.5%). In 19 (9.5%) of the procedures, the drain caliber was not noted.

There was a need for an additional intervention (nephrostomy or other procedure) in 74 (36.6%) of the 201 cases. The reasons for the additional intervention were as follows: the need to reposition the catheter, in 42 (20.9%) of the cases; the need for a larger caliber in order to insert a double J stent, in ten (5.0%); nephrostomy malfunction, in seven (3.5%); the need to replace the catheter because of the time since nephrostomy, in six (3.0%); infection, in five (2.5%); and unreported in four (2.0%).

**Figure 5:**
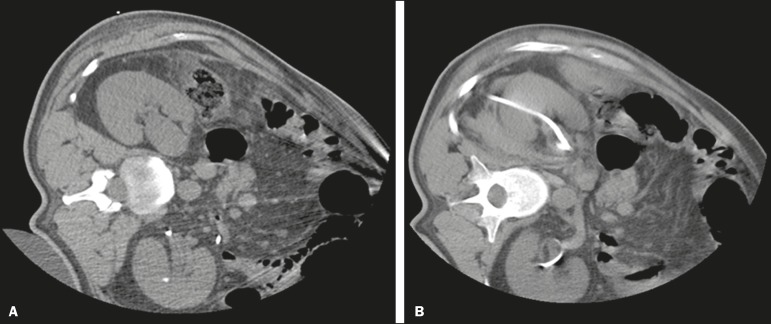
CT-guided percutaneous nephrostomy of the right kidney, by the Seldinger technique. **A:** Pre-procedure axial slice. **B:** Final follow-up CT showing stabilization of the bleeding.

Complications occurred in 19 (9.5%) of the cases: perirenal hematoma (Figure 5), in nine patients (4.5%); local infection, in one (0.5%); bleeding from the catheter, due to a pseudoaneurysm (as seen on arteriography), in one (0.5%); late hemorrhagic complications (Figure 2), resulting in death, in one (0.5%); and failed catheter placement, in 7 (36.8%)-due to insufficient dilatation, in 4 (2.0%) and due to patient agitation, resulting in the need to interrupt the procedure, in 3 (1.5%).

As can be seen in Table 1, the occurrence of complications after percutaneous nephrostomy was not found to show a statistically significant correlation with previous cancer treatment, the puncture technique used, the drain caliber, or the degree of hydronephrosis.

**Table 1:** Analysis of the occurrence of complications after CT-guided percutaneous nephrostomy, in relation to the variables studied, in cancer patients.

	Complications		
Variable	Yes	No	*P*
Previous cancer treatment			0.225
Yes	17	172	
No	2	10	
Technique			0.267
Seldinger	8	132	
Trocar	2	2	
Drain caliber			0.267
6 Fr	0	1	
10 Fr	9	131	
12 Fr	2	38	
14 Fr	0	1	
Grade of dilatation			0.046
0	9	60	
1	0	15	
2	5	45	
3	4	43	
4	1	17	

## DISCUSSION

The way in which urology patients are approached has been dramatically altered by advances and technical refinements in the field of interventional radiology. The expansion of the indications for percutaneous nephrostomy was possible only after its safety and efficacy as a means of accessing the renal collecting system had been established^(^^15^^)^.

In some studies, such as that conducted by Martin et al.^(^^16^^)^, it has been recommended that percutaneous nephrostomy be performed without pre-procedure analysis of the coagulation profile. However, we disagree with that approach, unless the situation is an absolute emergency. Because the kidneys are highly vascularized, needle puncture and dilatation of the urinary tract in a patient with a bleeding disorder can result in massive hemorrhage that is often difficult to control.

In our study sample, there was a predominance of elderly patients, the mean age being 63 years, and percutaneous nephrostomy was used mainly as a method of diverting the urinary collecting system to bypass neoplastic obstructions. Those data are in agreement with the findings of Farrell et al.^(^^17^^)^.

In 32.4% of our cases, the dilatation was classified as mild. That underscores the fact that the indications for nephrostomy include conditions other than obstructive nephropathy. Lee et al.^(^^18^^)^ showed that it is also useful for the treatment of urinary fistulas, infusion of chemotherapeutic or antibiotic substances, and decompression of perirenal collections, such as abscesses. In some cases, an additional intervention/nephrostomy can be justified even when there is no significant dilatation of the renal collecting system. Such intervention was necessary in 36.6% of the procedures evaluated in the present study. Farrell et al.^(^^17^^)^ and Lee et al.^(^^18^^)^ showed that it is possible to perform an additional intervention when there is displacement or obstruction of the catheter, which takes on an urgent character when there is a clinical profile suggestive of infection, as was observed in 5 (2.5%) of the patients in our sample, in whom additional intervention was indicated because of an infectious etiology.

Most studies report that percutaneous nephrostomy has a complication rate of approximately 10%, mortality rates ranging from 0.05 to 0.3%^(^^19^^,^^20^^)^. In the present study, we found that complications after percutaneous nephrostomy were not significantly associated with the type of previous cancer treatment, the technique employed, the caliber of the drain used, or the degree of dilatation of the renal collection system prior to the procedure. That is due in part to the safe nature of the procedure, with respect to the technical details, which are subject to the professional experience of the interventional radiologist, without independently raising the risk of complications^(^^21^^)^. From that perspective, it is also possible to infer that the reason that neither the degree of dilatation of the renal collecting system nor the type of previous cancer treatment were significantly associated with complications in the present study was because percutaneous nephrostomy has high success rates when correctly indicated and guided by CT. Nevertheless, complications were observed in 9.5% of the cases, corroborating data in the literature, and death occurred in one cancer patient (0.5%) who had multiple comorbidities and pronounced blood dyscrasia.

Other significant complications of percutaneous nephrostomy occurring in the present study, including perirenal hematoma, were treated conservatively. Slight transient bleeding, typically from veins or smaller vessels, is common after percutaneous nephrostomy. However, it is important to note that severe hemorrhage requiring transfusion or another urgent intervention is reported in 1-3% of patients undergoing percutaneous nephrostomy^(^^20^^)^.

As a treatment for a dilated, obstructed renal collecting system, percutaneous nephrostomy is successful in 98-99% of cases. As would be expected, the reported success rate is lower (85-90%) in patients with a non-dilated renal collecting system^(^^19^^)^. Kalogeropoulou et al.^(^^22^^)^ and Gamal et al.^(^^23^^)^ reported a certain amount of difficulty in performing the procedure in such patients. In the present study, failure due to a lack of hydronephrosis was observed in only 2% of the cases. With adequate training in the latest technological advances, the absence of significant hydronephrosis should not be considered a limiting factor for percutaneous nephrostomy.

## CONCLUSION

CT-guided percutaneous nephrostomy has become a routine procedure in the practice of the interventional radiologist. The results of the present study indicate that the procedure is effective in cancer patients, with success rates and complication rates similar to those observed in the general population.
